# Targeting miR-155 to Treat Experimental Scleroderma

**DOI:** 10.1038/srep20314

**Published:** 2016-02-01

**Authors:** Qingran Yan, Jie Chen, Wei Li, Chunde Bao, Qiong Fu

**Affiliations:** 1 Department of Rheumatology, Renji Hospital, School of Medicine, Shanghai Jiaotong University, Shanghai 200001, China; 2 Xijing Hospital, The Fourth Military Medical University, Department of Dermatology, Xi’an 710032, China

## Abstract

Scleroderma is a refractory autoimmune skin fibrotic disorder. Alterations of microRNAs in lesional skin could be a new approach to treating the disease. Here, we found that expression of miR-155 was up regulated in lesional skin tissue from patients with either systemic or localized scleroderma, and correlated with fibrosis area. Then we demonstrated the potential of miR-155 as a therapeutic target in pre-clinical scleroderma models. MiR-155^−/−^ mice were resistant to bleomycin induced skin fibrosis. Moreover, topical antagomiR-155 could effectively treat mice primed with subcutaneous bleomycin. In primary skin fibroblast, miR-155 silencing could inhibit collagen synthesis function, as well as signaling intensity of two pro-fibrotic pathways, Wnt/β-catenin and Akt, simultaneously. We further showed that miR-155 could regulate the two pathways via directly targeting casein kinase 1α (CK1α) and Src homology 2-containing inositol phosphatase-1 (SHIP-1), as previous reports. Mice with miR-155 knockout or topical antagomir-155 treatment showed inhibited Wnt/β-catenin and Akt signaling in skin upon bleomycin challenge. Together, our data suggest the potential of miR-155 silencing as a promising treatment for dermal fibrosis, especially in topical applications.

Scleroderma is an autoimmune disorder characterized by excessive collagen deposition in dermis. It can be localized in morphea^1^ and systemic in systemic sclerosis (SSc), where fibrosis and failure of inner organs can be present^2^. Fibrosis is an entity poorly responding to approve treatments for scleroderma and new therapies are in great need.

MicroRNA (miRNA) is a promising treatment target in multiple diseases. These noncoding 22- or 23-nucleotide RNAs can induce silencing complex by recognizing specific site on 3′ UTR of target mRNAs^3^. Dysregulation of miRNAs has emerged in fibroblasts from SSc patients, such as miR-21, miR-92, miR-29, miR-150, miR-7, miR-30b, and miR-196a^4^,^5^,^6^,^7^,^8^. Several of the miRNAs show potentials for therapeutic application, for they are altered upon anti-fibrotic drugs in animal models^4^,^7^,^9^. Among them, miR-155 is found up-regulated in skin fibroblasts from patients with SSc^8^, while its clinical significance and role in treatment are still not clear.

MiRNA has shown therapeutic value in treatment of experimental skin fibrosis through intraperitoneal let-7^10^. However, compared with systemic administration, local application of miRNAs is a favorable option for localized morbidities. In fact, intradermal injection of miR-21 has shown therapeutic benefits to human psoriasis skin graft^11^. Other agonist or antagonist of miRNAs have been injected into coronary artery^12^, brain^13^ or injured muscle^14^, and successfully treated the diseases in mouse models. For scleroderma patients, topical treatment can be less traumatic than intradermal injection. Hence targeting miRNA topically to treat scleroderma would be an interesting discovery.

Here in this work, we show that miR-155 expression was elevated in skin tissue from patients with localized and systemic scleroderma as well as from experimental skin fibrosis model. Both local and systemic miR-155 silencing in further *in vivo* study could remarkably attenuate bleomycin induced dermal fibrosis. Therefore, miR-155 could be a potential treatment target for scleroderma, especially via topical administration.

## Results

### MiR-155 was up-regulated in skin tissues from patients with scleroderma and experimental skin fibrosis model

**Figure 1:**
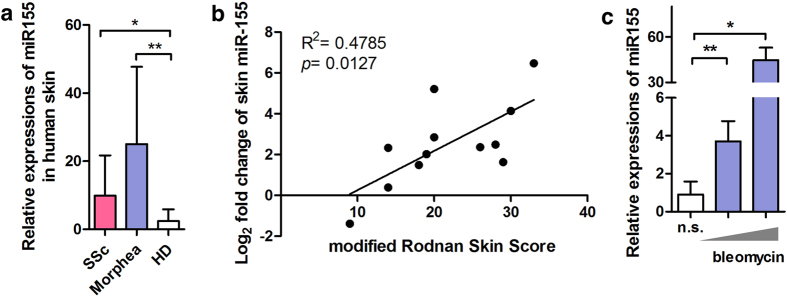
MiR-155 was up-regulated in fibrotic skin tissues. (**a**) Expression of miR-155 in skin tissues from patients with SSc (n = 12) and morphea (n = 7) as well as healthy donors (HD, n = 9). *P < 0.05, **P < 0.01, ANOVA. (**b**) Skin miR-155 was biologically related with extent of skin sclerosis in SSc patients. Linear regression. (**c**) Expression of miR-155 in skin tissues from C57/BL6 mice injected with bleomycin (1 mg/ml and 5 mg/ml, n = 3 per group) subcutaneously. *P < 0.05, **P < 0.01, ANOVA.

Compared with healthy donors, miR-155 expression was up-regulated in skin from SSc patients; and it was even higher in the skin from morphea patients (Fig. 1a). Moreover, miR-155 expression in lesional skin showed a strong positive correlation with the extent of skin involvement in SSc patients (Fig. 1b). In animal study, we injected male C57BL/6 (B6) mice with bleomycin subcutaneously, which would cause dramatic collagen deposition and thickening of dermis (Supplementary Fig. S1). Quantitative RT-PCR showed up-regulation of miR-155 in fibrotic skin related with dosage of bleomycin (Fig. 1c).

### MiR-155^−/−^ resisted bleomycin induced skin fibrosis

**Figure 2:**
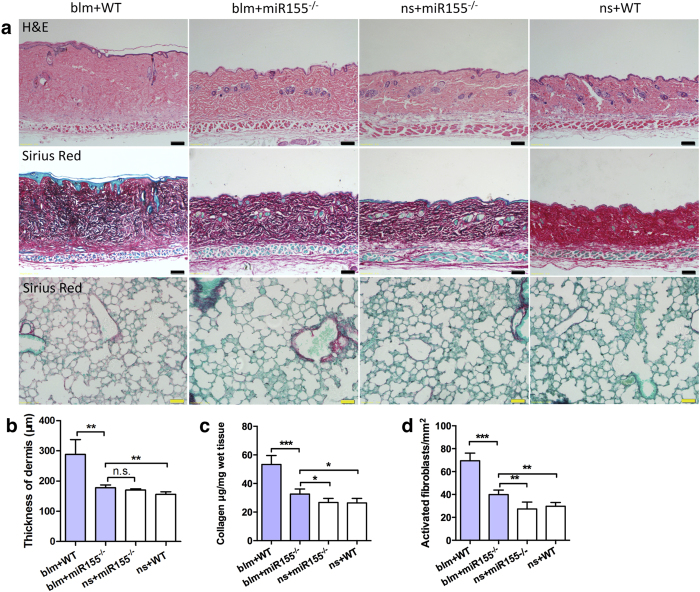
MiR-155^−/−^ resisted bleomycin induced skin fibrosis. From day 0, miR-155^−/−^ and wild type C57/BL6 (WT, B6) mice were injected subcutaneously with either a bleomycin (blm) solution (5 mg/ml) or saline (n.s.) every other day. Mice were sacrificed on day 21. (**a**) Representative skin and lung sections with H&E or Sirius red staining from each group. For Sirius red staining, red parts represent stained collagen and green parts represent total protein. Bars, 100 μm in skin sections and 50 μm in lung sections. (**b**) Thickness of dermis. N = 5 per group. **P < 0.01, ANOVA. (**c**) Collagen content of lesional skin measured by Sircol assay. N = 5 per group. *P < 0.05, ***P < 0.001, ANOVA. (**d**) Density of activated fibroblasts (α-SMA+) in each skin sample. N = 5 per group. *P < 0.05, ANOVA.

We injected bleomycin subcutaneously and observed significant skin and lung fibrosis in wild type C57/BL6 (B6, WT) mice as reported previously^15^. However, miR-155^−/−^ mice showed less fibrosis in dermis when challenged with bleomycin (Fig. 2a). Skin thickness and collagen content measurements showed statistically significant difference between miR-155^−/−^ and WT mice (Fig. 2b,c). Similar results were observed in collagen deposition in lung tissues (Fig. 2a). Besides, α-smooth muscle actin (α-SMA) positive activated fibroblasts were significantly decreased in skin from miR-155^−/−^ mice (Fig. 2d).

### Topical antagomiR-155 effectively treated bleomycin induced skin fibrosis

**Figure 3:**
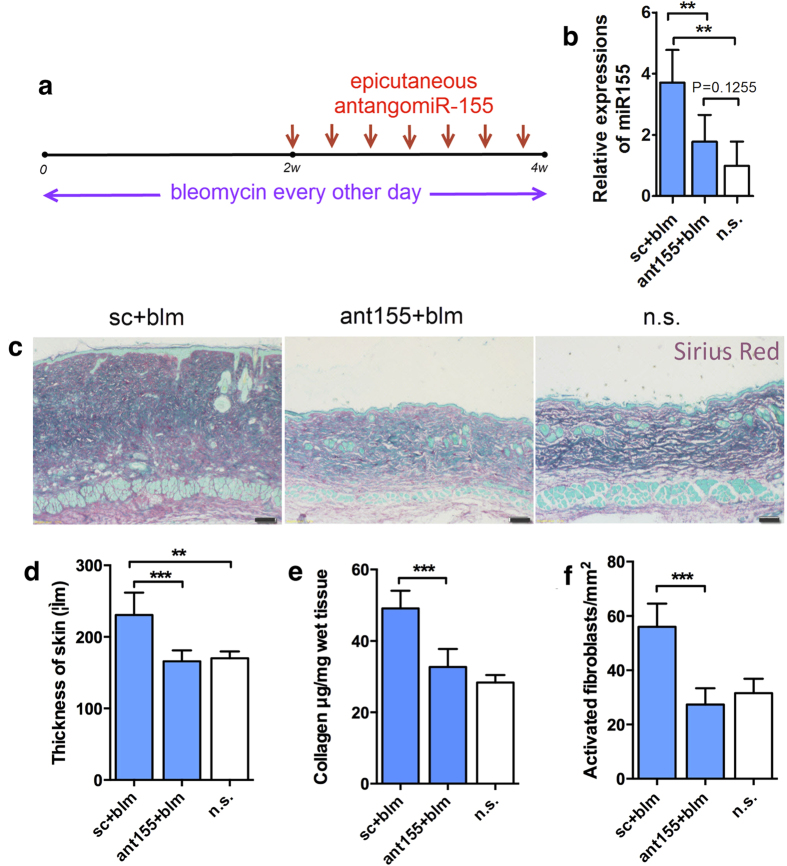
Topical antagomiR-155 effectively treated bleomycin induced skin fibrosis. (**a**) C57/BL6 mice were injected subcutaneously with either a bleomycin (blm) solution (1 mg/ml) or saline (n.s.). Topical antagomiR-155 (ant155) or scramble control (sc) was applied every other day from day 15. Mice were sacrificed on day 28. (**b**) MiR-155 expression in treated skin area on day 28. N = 7 per group. **P < 0.01, ANOVA. (**c**) Representative skin sections stained with Sirius red from each group. Red parts represent stained collagen and green parts represent total protein. Bars, 100 μm. (**d**) Thickness of dermis. N = 7 per group. **P < 0.01, ***P < 0.001, ANOVA. (**e**) Collagen content of lesional skin measured by Sircol assay. N = 7 per group. ***P < 0.001, ANOVA. (**f**) Density of activated fibroblasts (α-SMA+) in each skin sample. N = 7 per group. ***P < 0.001, ANOVA.

We first demonstrated that transcutaneous absorption of topical antagomiR-155 worked in C57/BL6 mice, as shown in the trace of conjugated Cy3 (Supplementary Fig. S2). Then we treated the mice with antagomiR-155 two weeks after preliminary bleomycin induction (The procedure is depicted in Fig. 3a). After two-week treatment, miR-155 expression in skin, but not in liver, bone marrow or blood cells, could be effectively down regulated by topical antagomiR-155 (Fig. 3b, Figure S3). No overt side effects were observed. Topical antagomiR-155 significantly decreased the dermal thickening (Fig. 3b,c), collagen deposition (Fig. 3d), as well as density of α-SMA+ activated fibroblasts in bleomycin challenged skin tissue (Fig. 3e).

### MiR-155 silencing inhibited collagen production in primary skin fibroblasts

**Figure 4:**
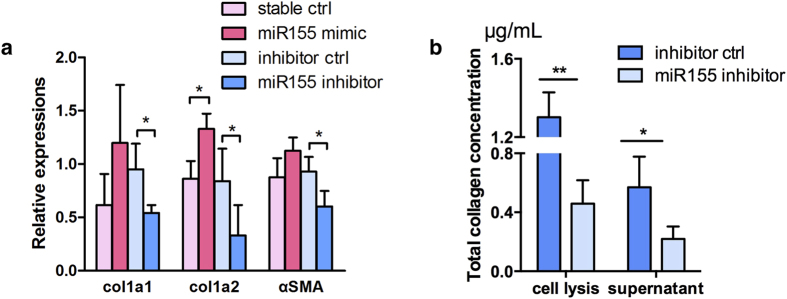
MiR-155 silencing inhibited collagen production in primary skin fibroblast with TGF-β stimulation. Data were from three independent experiments. (**a**) MiR-155 was related with expression of type I collagen and α-SMA genes in skin fibroblasts from C57/BL6 mice. *P < 0.05, ANOVA. (**b**) Collagen concentrations in supernatant measured by Sircol assay. *P < 0.05 **P < 0.01, non-paired student’s *t* test.

We isolated primary mouse skin fibroblasts and transfected them with miR-155 mimic or inhibitor, which successfully regulated miR-155 expression 24 hours after transfection (Supplementary Fig. S4). The mRNA expressions of type I collagen were elevated with miR-155 mimic and decreased with miR-155 inhibitor, and similar changes were observed on α-SMA (Fig. 4a). Further, miR-155 inhibitor could also remarkably decreased collagen released to the supernatant (Fig. 4b).

### MiR-155 regulated Wnt/β-catenin and Akt signaling *in vitro*

**Figure 5:**
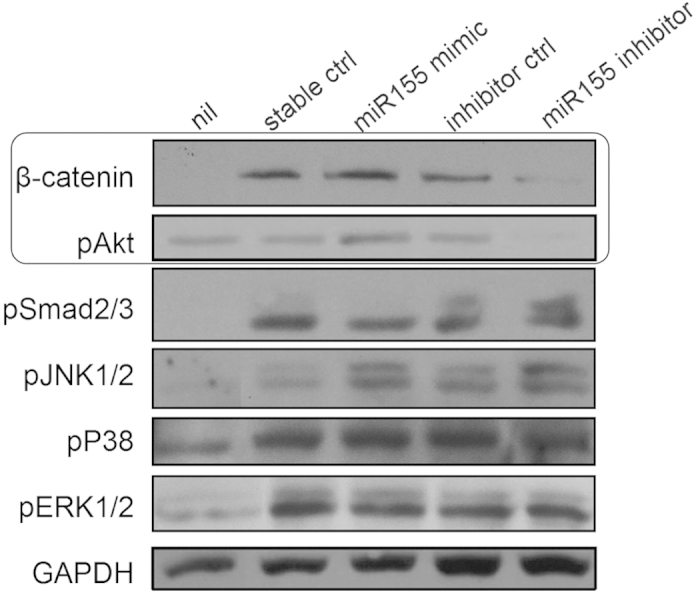
MiR-155 regulated Wnt/β-catenin and Akt signaling *in vivo*. Pro-fibrotic signal pathways were analyzed in mouse primary skin fibroblast 1 hour after TGF-β stimulation, with transfection of miR-155 mimic or inhibitor.

Several major signaling pathways have been found to promote fibrosis and SSc development^2^. We screened these pathways using western blot analysis in primary skin fibroblast challenged with TGF-β. Among these pathways, we noticed that β-catenin and Akt signaling intensity could be regulated by miR-155. MiR-155 mimic could strongly decrease the degradation of β-catenin and increase the phosphorylation of Akt, while miR-155 inhibitor did the opposite to the two pathways (Fig. 5).

### MiR-155 regulated Wnt/β-catenin and Akt signaling by directly targeting CK1α and SHIP-1, respectively

**Figure 6:**
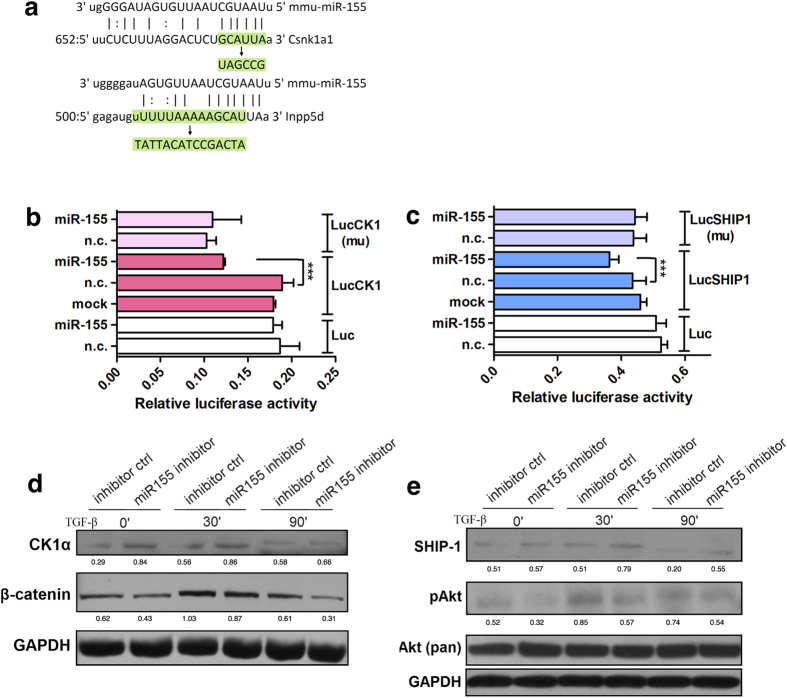
MiR-155 regulated Wnt/β-catenin and Akt signaling in primary fibroblast by targeting CK1α and SHIP-1 with TGF-β stimulation. (**a**) Depiction of mouse CK1α (*csnk1a1*) and SHIP-1 (*inpp5d*) mRNA 3′ UTR sequence alignment with miR-155 sequence. Mutant sites are marked green. (**b**) MiR-155 bound directly to the 3′ UTR of mouse CK1α mRNA. A luciferase reporter assay was co-transfected with miR-155 mimic or normal control (n.c.) into HEK293 cells. ***P < 0.001, ANOVA. (**c**) Similarly, miR-155 bound directly to the 3′ UTR of mouse SHIP-1 mRNA. ***P < 0.001, ANOVA. (**d**) MiR-155 inhibitor up-regulated CK1α and degradation of β-catenin spontaneously. The numbers below lanes represent optic density ration to GAPDH. (**e**) Similarly, miR-155 regulated protein levels of SHIP-1 and phosphorylation of Akt. The numbers below lanes represent optic density ration to GAPDH.

We conducted a bio-informatics search and identified casein kinase 1α (CK1α), a negative modulatory protein on β-catenin pathway, as a predicted target of miR-155 in human and mouse. Meanwhile we identified a negative regulator on Akt signal pathway, Src homology 2-containing inositol phosphatase-1 (SHIP-1), as another target of miR-155 (Fig. 6a). Then we developed a luciferase reporter construct consisting of CK1α 3′-UTR miR-155 binding region (LucCK1), and used a mutated construct (LucCK1mu) and vehicle plasmid (Luc) as controls (mutated sequences are depicted in Fig. 5a). MiR-155 mimic significantly decreased luciferase activity in HEK293cells transfected with LucCK1 reporter, compared with cells that transfected with control vectors (Fig. 6b), which suggested that CK1α is a direct target of miR-155. The direct interaction between miR-155 and 3′-UTR of mouse SHIP-1 mRNA was similarly demonstrated (Fig. 6c). Further Western blot analysis showed that miR-155 silencing could increase both CK1α and SHIP-1 protein levels, with inhibited β-catenin degradation and Akt phosphorylation simultaneously (Fig. 6d,e).

### Both systemic and topical miR-155 targeting regulated Wnt/β-catenin and Akt signaling *in vivo*

**Figure 7:**
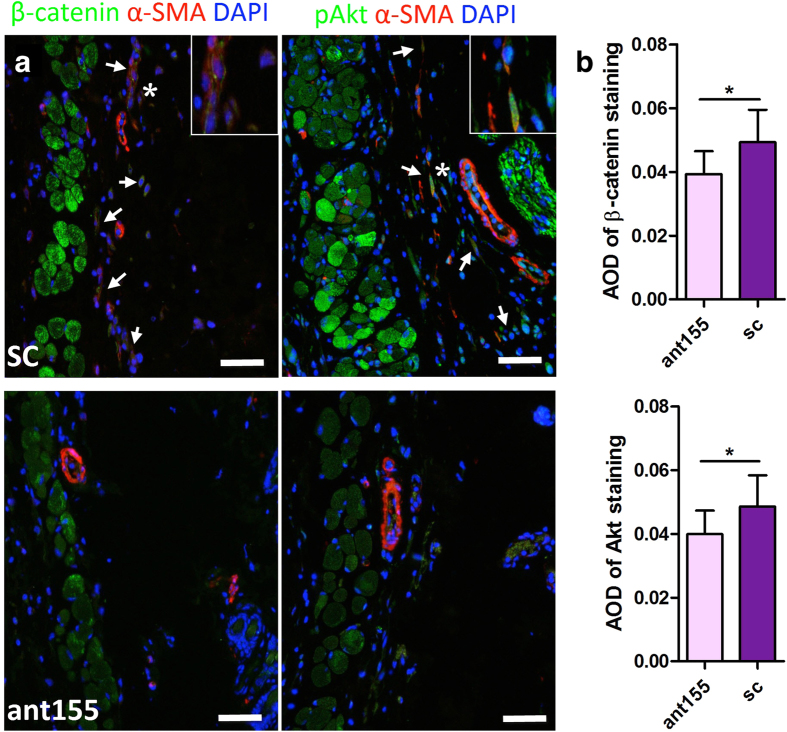
Topical antagomiR-155 regulated Wnt/β-catenin and Akt signaling pathways in skin tissue. (**a**) Representative skin sections stained with β-catenin, pAkt, and α-SMA. Arrows indicate fibroblasts with up-regulated β-catenin or pAkt. Zoomed areas are marked with asterisks. Bars, 20 μm; epidermal side of each sample was on the right. (**b**) Average optic density (AOD) values of β-catenin and pAkt staining from the dermal layer between epidermis and muscle. *P < 0.05, Mann-Whitney test.

Protein level of β-catenin and pAkt both showed decreasing trend in miR-155^−/−^ mouse skin tissue, compared with WT mice (Supplementary Fig. S5). Topical antagomiR-155 application lead inhibited staining of β-catenin and phosphorylated Akt in multiple cell sets from skin tissue, including but not limited to fibroblasts (Fig. 7a). Average optic density (AOD) value of dermal layer from treatment group was significantly less than control (Fig. 7b). These *in vivo* findings were consistent with what we observed in the *in vitro* study above.

## Discussion

SSc is a heterogeneous disease whose pathogenesis is characterized by three hallmarks: excessive deposition of extracellular matrix, small vessel vasculopathy and production of autoantibodies^2^. Though the clinical manifestations of SSc vary, most of the patients have skin thickening and variable involvement of internal organs. Compared with vasculopathy or autoimmunity, fibrosis of skin and other organs still lacks approved treatments. Tyrosine kinase and TGF-β inhibitors have shown potential anti-fibrotic effects in experimental research recently; however none of them succeed in randomized clinical trials^16^. Here we describe that miRNA could be a novel potential treatment for fibrosis.

Morphea is also a disorder characterized with excessive collagen deposition in dermis or subcutaneous tissue^1^. However, unlike SSc, it lacks vasculopathy features such as sclerodactyly, Raynaud phenomenon, nailfold capillary changes, and telangiectasias^17^. In our study, miR-155 was upregulated both in SSc and morphea skin samples. This indicated that the work of miR-155 in scleroderma might not depend on vascular injury. On the other side, the tendency that patients with larger fibrosis area or inner organ sclerosis had higher miR-155 expression in their skin further supported possible relation of miR-155 with fibrosis.

Recent data indicated that the miR-155 expression is up-regulated in many inflammatory fibrosis syndromes other than scleroderma, including idiopathic pulmonary fibrosis^18^,^19^, cystic fibrosis^20^,^21^ and alcoholic/nonalcoholic liver fibrosis^22^,^23^,^24^, as well as in animal models of these diseases^20^,^22^,^24^,^25^,^26^. Besides, loss of miR-155 in mice can significantly inhibit pressure-overload^27^ or diabetes^28^ induced cardiac fibrosis and remodeling, suggesting the potential of miR-155 as a treatment target in fibrotic conditions.

In this study, targeting miR-155 could inhibit Wnt/β-catenin and Akt pathways, which are necessary for fibrosis development. Our data indicate miRNA as a novel approach to touch the two pathways simultaneously. As one of the most well accepted pro-fibrotic signaling pathways, Wnt/β-catenin pathway is demonstrated to be involved in SSc development and the experimental models^29^,^30^,^31^. Meanwhile, Akt is also activated in SSc fibroblasts, and blocking Akt by siRNA, small molecular inhibitor^32^ or its upstream protein^33^ can treat experimental skin fibrosis effectively. Moreover, the two pathways can crosstalk with canonical TGF-β signaling; they both can be activated by canonic pro-fibrotic cytokine TGF-β in fibroblast^34^ and other cell types^35^.

CK1α is a serine/threonine kinase leading phosphorylation and degradation of multiple components of β-catenin pathway, and can be up-regulated by β-catenin as a negative feedback^36^. Previous study on human liposarcoma has demonstrated that miR-155 impacts β-catenin signaling through directly targeting CK1α^37^. To our knowledge, it is the first time to show the potential role of CK1α in scleroderma treatment. While another member of Casein kinase family, CK II, has also recently emerged as a possible therapeutic target for scleroderma^38^.

Similarly, SHIP-1 is a phosphatase that can abolish phosphorylation of Akt, which promote cell proliferation and survival^39^,^40^. SHIP-1 has also been proved as a direct target of miR-155^41^. In fact, regulation of SHIP-1 by miR-155 is critical to autoimmunity or inflammation in animal studies, such as arthritis^42^ and lupus^43^. Present researches have also shed some light on SHIP-1 and fibrosis. SHIP-1 is essential for proliferation, survival, migration and collagen production of fibroblasts^44^,^45^,^46^; and SHIP-1 deficiency attenuates airway fibrosis in allergy mouse model^47^.

Our study revealed a potentially novel treatment approach to target miRNA. To our knowledge, this study first reports that a cholesterol-conjugated antagomiR has succeeded to treat skin lesion epicutaneously. It is plausible to hypothesize that epicutaneous antagomiR-155 could be especially beneficial to patients with local scleroderma such as morphea. Further *in vivo* study on human skin tissue is warranted.

## Methods

### Patients and Healthy Control Subjects

Skin specimens were obtained from paraffin embedded tissues of biopsy of patients with systemic sclerosis (SSc) or localized scleroderma (morphea). All patients with SSc met the 2013 classification criteria for SSc by ACR/EULAR. Modified Rodnan skin score (mRSS) was calculated to assess skin involvement for each patient with SSc according to previous report^48^. Demographic information of SSc patients is shown in Table S1. Skin tissues from healthy donors were collected from healthy volunteers during plastic surgery. Then the tissues were also embedded with paraffin. All skin samples were collected from Renji Hospital, Shanghai Jiaotong University and Xijing Hospital, The Fourth Military Medical University.

### Ethical Consideration

This study was conducted according to the principles expressed in the Declaration of Helsinki. Informed consent was obtained from all subjects. The Shanghai study was approved by the Institutional Review Board of Renji Hospital. The studies of the Xi’an samples were approved by the Research Ethics Committee of Xijing Hospital, The Fourth Military Medical University.

### Animals

Male miR-155 knockout (B6.Cg-Mir155tm1.1Rsky/J) and wild type^49^ C57BL/6 (B6) mice were kindly provided by Laboratory of Molecular Rheumatology, Institute of Health Science, Chinese Academy of Science and Shanghai Jiaotong University School of Medicine. All mice were purchased from Jackson Laboratories (Bar Harbor, Maine, USA). Both strains were bred in-house in a pathogen-free facility. Animal experiments were carried out according to institutionally approved protocols of the Animal Care and Use Committee of Shanghai Jiaotong University, Shanghai, China.

### Induction and topical treatment of Experimental Fibrosis

From day 0, miR-155^−/−^ or B6 mice (8-week-old) were injected subcutaneously in a 1.5 × 1.5 cm area on the back with 100 μl bleomycin solution or normal saline every other day. On day 21, all the mice were sacrificed by cervical dislocation.

For topical treatment, B6 mice were first injected subcutaneously with bleomycin every other day for two weeks; from day 15, the mice were applied antagomiR-155 or scramble control (RiboBio Co., Ltd., Guangzhou, China) epicutaneously every other day for another two weeks with continuous bleomycin injection. The antagomiR-155 and scramble control were 3′-cholesterol and 2′-OMe modified and dissolved in 95% acetone at concentration of 0.67 nmol/ml. Each mouse was administrated 2.6 nmol of antagomiR-155 or scramble control on the lesional skin area each time. The mice were sacrificed on day 28.

### Tissue Fibrosis assessment

For histologic assessment, mouse skin samples were fixed and stained with hematoxylin and eosin and and Sirius red (Chondrex Inc., WA, USA) staining according to the manufacturer’s instructions. Thickness of dermis of each mouse was calculated as mean value of two distinct Sirius red staining sections, with five measurements at different positions in each section. Skin collagen content was measured using Sircol collagen dye-binding assay (Biocolor, Belfast, Northern Ireland) according to the instruction of manuscript, where each mouse was sampled by skin punch biopsies (6-mm in diameter) from the bleomycin injection site.

For skin fibroblast counting, α-SMA positive fibroblast (the staining is detailed in the next passage) was counted as the mean value of two distinct sections for each mouse. Each section included five random fields at 400 times magnification.

### Immunohistochemistry and immunofluorescent staining of mouse skin tissue

The staining was performed in paraformaldehyde-fixed skin sections. Samples were incubated with mouse-anti-α-SMA, rabbit-anti-pAkt, rabbit-anti-β-catenin antibodies.

For immunohistochemistry assay, the sections were secondly stained with horseradish peroxidase (HRP)-labeled goat-anti-rabbit IgG antibody. The immune reactivity was detected with 3, 3′-diaminobenzidine kit (BD Bioscience, CA, USA).

Similarly for immunofluorescence assay, the sections were secondly stained with Alex488 labeled goat-anti-mouse IgG or CY3 labeled goat-anti-rabbit IgG antibodies. Each fluorescent staining was recorded by the OlyVIA system (Olymphus, Southend-on-Sea, UK) under one condition. Average optic density (AOD) of each sample was calculated as the mean value of two distinct fields at 100 times magnification by using Image Pro Plus 6.0 (Media Cybernetics, Rochville, MD, USA). Skin layers only between muscle and epidermis were counted in AOD calculation.

### Primary skin fibroblast isolation and culture

We digested fresh skin tissue from juvenile B6 mice (younger than one week, 6 mice for one time) with 0.1% dispase II overnight, removed departed epidermis, treated the dermis with 0.1% collagenase I, and filtered digested cell suspension with nylon membrane. Then the cells were cultured with Dulbecco’s modified eagle medium (DMEM) supplemented with 10% fetal calf serum and passage 3 to 7 was used for experiments. Supernatant collagen concentration was detected by Sircol collagen dye-binding assay according to manuscript (Biocolor).

### Transfection experiments

Cells were transfected with miR-155 mimic, miR-155 inhibitors, or scrambled miRNA controls (named “negative control” and “inhibitor control”) (Applied Biosystems, Thermo Fisher Inc., MA, USA) at a final concentration of 40 nM with the use of Lipofectamine 2000 reagent (Invitrogen, Thermo Fisher Inc.). After 24 hours of transfection, cells were stimulated with TGF-β (10 ng/ml).

### Quantitative PCR

Total RNA from cultured cell was isolated using the TRIzol reagent; RNA from paraffin sections was isolated using RNeasy PPFE kit (Qiagen, Hilden, Germany). The reverse transcription kit (Qiagen) was used for cDNA preparation. TaqMan miRNA assay (Applied Biosystems) was used for determination of the expression level of mouse miR-155 (MS00001701) and human miR-155 (MS00003605). The expression of U6B small nuclear RNA was used as endogenous control. SYBR Green Master Mix (Applied Biosystems) was used for determination of mRNA expression of mouse col1a1, col1a2, and α-SMA. The expression of GAPDH was used as endogenous control (primer sequences seen in Table S2). Each sample in the chain reaction was amplified in triplicate.

### Western blot analysis

Primary mouse skin fibroblasts were lysed by RIPA solution and fresh tissue was lysed using T-PER reagent with proteinase and phosphatase inhibitor (Thermo Fisher). After gel electrophoresis and electrotransferation, proteins were detected with antibodies against SHIP-1, CK1α, pAkt, Akt (pan), β-catenin, pErk, pJNK, pSmad2/3, pp38 and GAPDH. HRP conjugated goat anti-rabbit or anti-rat secondary antibodies were used. Semiquantitative analysis based on densitometry was performed using Image J software (National Institute of Health, Bethesda, MD, USA).

### Luciferase Activity Assay

The mouse CK1α miRNA target site and its mutation were amplified by primers; the target site was predicted by bioinformatics database including miRBase, PicTar and Target Scan Human. These PCR products were both cloned downstream of the luciferase gene in psiCHECK-2 luciferase vector (Promega, WI, USA), and the constructs were named “Luc-CK1α” and “Luc-CK1α (mu)”. These constructs were transfected together with miR-155 mimic or scrambled miRNA into HEK293 cells. Luciferase activity was measured using the Dual-Luciferase Reporter Assay (Promega) 24 h after transfection. Each treatment was performed in triplicate.

Similarly, we made “Luc-SHIP1” constructs by cloning SHIP1 target site into psiCHECK-2 luciferase vector. The target site was amplified from a commercial plasmid miReport SHIP-1 3′ UTR (Addgene Inc., MA, USA). Then “Luc-SHIP1 (mu)” constructs were made. Sequences of the primers above are shown in Table S3.

### Statistical analysis

All continuous variables were expressed as means ± SD. Comparisons between two groups were tested for statistical significance with unpaired *t* test or Mann-Whitney *U* test, as appropriate. Comparison among three or more groups was performed with analysis of variance (ANOVA) followed by Bonferroni correction. Correlation between two groups of continuous variables was analyzed with linear regression. All statistical analysis was performed using SAS 11.0 (SAS Institute Inc., Cary, NC, USA).

## Additional Information

**How to cite this article**: Yan, Q. *et al.* Targeting miR-155 to Treat Experimental Scleroderma. *Sci. Rep.* **6**, 20314; doi: 10.1038/srep20314 (2016).

## Supplementary Material

Supplementary Information
