# Corrigendum: Targeting miR-155 to Treat Experimental Scleroderma

**DOI:** 10.1038/srep21941

**Published:** 2016-03-04

**Authors:** Qingran Yan, Jie Chen, Wei Li, Chunde Bao, Qiong Fu

Scientific Reports 6: Article number: 20314;10.1038/srep20314 published online: 02012016; updated: 03042016

The Acknowledgements section in this Article is incomplete.

“The authors would like to thank all the laboratory coordinators from Shanghai Institute of Rheumatology: Ms. Ye Ping, Ms. Song Rui, Ms. Wang Zhenni and Mr. Zhu Haoming. Dr. Qu Bo and Dr. Han Xiao from Institute of Health Sciences, Chinese Academy of Sciences and Shanghai Jiao Tong University School of Medicine provided kind support on data analysis and animal experiments. Dr. Xiao Chun-yuan, Dr. Zhou Ying-ying and Ms. Sun Shu-hui from Institute of Rheumatology, Shanghai Renji Hospital helped in laboratory techniques.”

should read:

“The authors would like to thank all the laboratory coordinators from Shanghai Institute of Rheumatology: Ms. Ye Ping, Ms. Song Rui, Ms. Wang Zhenni and Mr. Zhu Haoming. Dr. Qu Bo and Dr. Han Xiao from Institute of Health Sciences, Chinese Academy of Sciences and Shanghai Jiao Tong University School of Medicine provided kind support on data analysis and animal experiments. Dr. Xiao Chun-yuan, Dr. Zhou Ying-ying and Ms. Sun Shu-hui from Institute of Rheumatology, Shanghai Renji Hospital helped in laboratory techniques.

This study was supported by the Natural Science Foundation of China (30901306 and 81471596), Shanghai Rising-Star Program (11QA1404200), Science and Technology Fund of Shanghai Jiaotong University School of Medicine and National key clinical center construction Program of China.”

