# Supplementary material for: Targeting miR-155 to Treat Experimental Scleroderma
Source: Sci Rep. 2016 Feb 1;6:20314. doi: 10.1038/srep20314 (PMC4734331; doi:10.1038/srep20314)
Supplement: Supplementary Information [file srep20314-s1.pdf]

Supplementary materials

Targeting miR-155 to Treat Experimental Scleroderma

Qing-ran Yan[1†], Jie Chen[1†], Wei Li[2], Chun-de Bao[1*] and Qiong Fu[1*]

[1] Renji Hospital, School of Medicine, Shanghai Jiaotong University, Department of Rheumatology, Shanghai Institute of Rheumatology, Shanghai 200001, China.

[2] Xijing Hospital, The Fourth Military Medical University, Department of Dermatology, Xi'an 710032, China

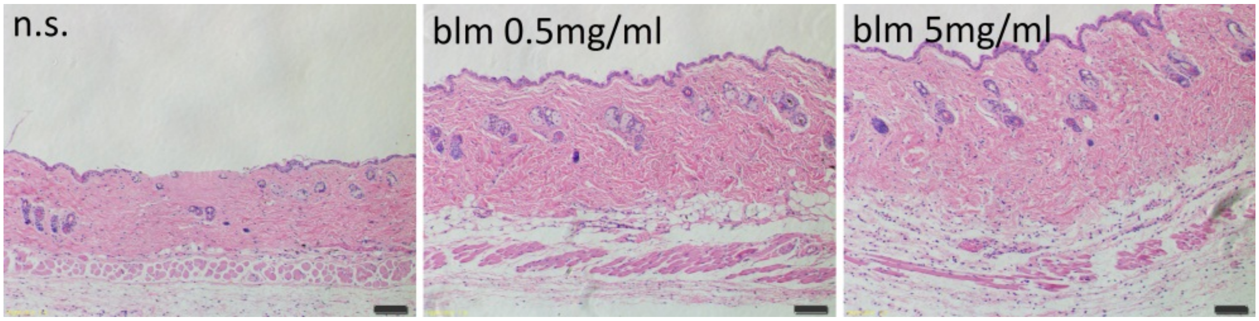

Figure S1. Representative H&E staining of skin samples from B6 mice injected with bleomycin or normal saline (n.s.). Bars represent 100 μm.

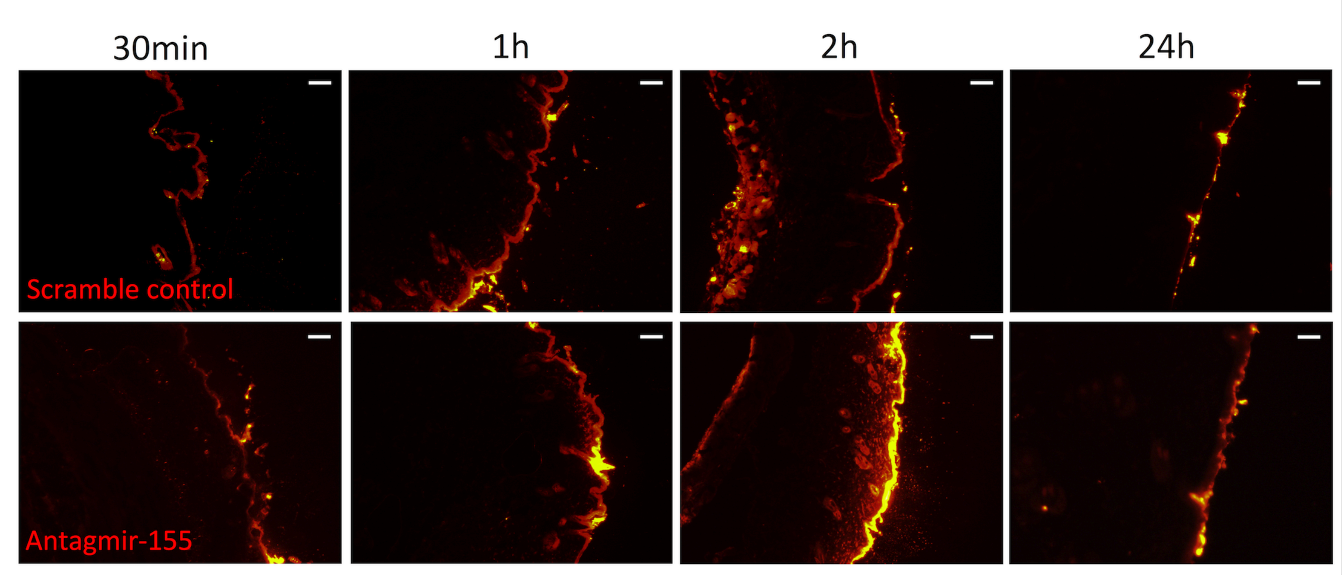

Figure S2. Trace of antagomiR-155 marked by conjugated Cy3 after topical administration. Bars,

100μm.

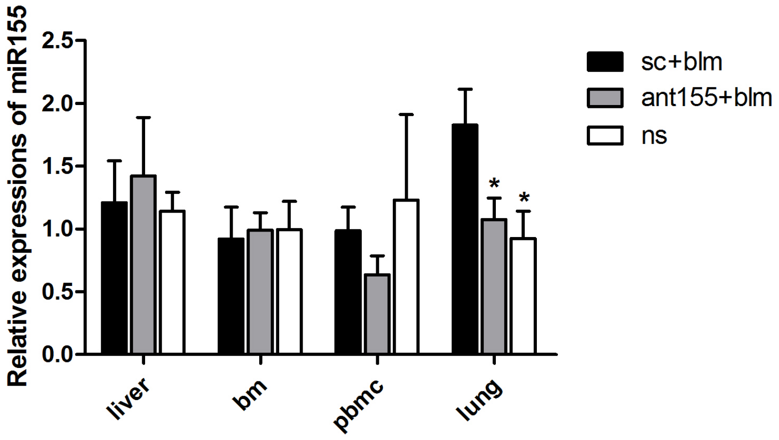

Fig. S3. Expression of miR-155 in multiple organs from B6 mice treated with antagomiR-155 epidermically for 2 weeks. Bleomycin was injected subcutaneously 2 weeks before the topical treatment. Data are represented as mean±SD. Statistics were calculated by non-paired student's *t*-test. * P<0.05. sc, scramble control; ns, normal saline; pbmc, peripheral blood mononuclear cells; bm, bone marrow. N=7 in each treatment.

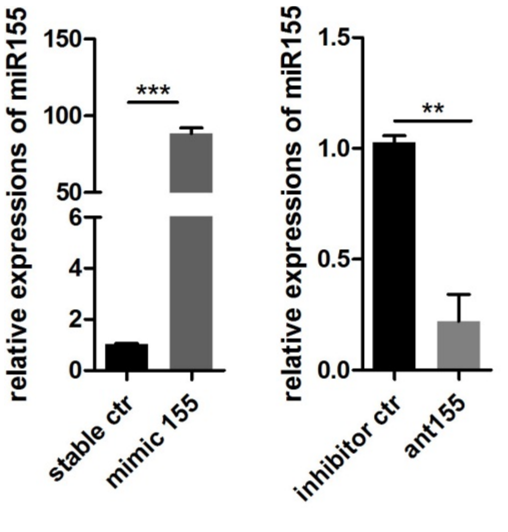

Figure S4. Expression of miR-155 in primary mouse skin fibroblast 24 h after transfection of miR-155 mimic or inhibitor. ** P<0.01, *** P<0.001, non-paired student's *t* test.

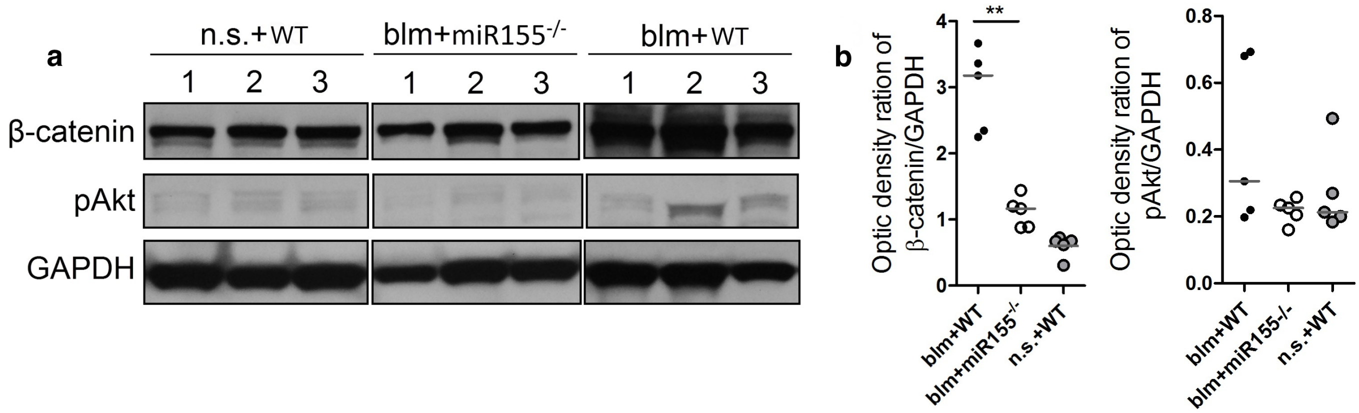

Figure S5. MiR-155$^{-/-}$ mice exhibited reduced β-catenin and Akt signaling after bleomycin induction. (A) Representative western blot results of β-catenin and pAkt in mouse skin tissues. (B) Semiquantitative optical density of the blotting. Each dot represents one mouse sample and horizontal lines represent media. **P<0.01, *U* test. blm, bleomycin; n.s., normal saline; WT, wild type.

Table S1. Clinical characters of the SSc patients at enrollment.

	sex	Age(yrs)	mRSS	systemic involvement*	Previous treatment†	disease duration (mo)
Pt1	F	39	26	GI	HCQ, PA, MP, herbs	14
Pt2	F	48	33	-	herbs	8
Pt3	F	51	18	-	Pred, LEF	36
Pt4	F	30	9	arthritis, GI	Pred, TwHF	15
Pt5	F	23	14	PF, PAH	Pred, colchicine, HCQ, PA, Cyc	6
Pt6	F	44	14	arthritis, GI	HCQ, TwHF, Pred	24
Pt7	F	68	28	GI,PF	Pred, herbs	120
Pt8	F	37	19	GI	herbs, HCQ, PA	12
Pt9	F	34	30	-	herbs, MP,	48
Pt10	F	36	20	GI, PF	herbs	3
Pt11	M	55	20	-	N/A	14
Pt12	F	11	29	-	herbs, HCQ, Pred	36

Each patient received evaluation including history, physical exam, laboratory test (at least include blood cell count, biochemistry, serum immunoglobulin and autoantibodies), chest image (X-ray or computer tomography). Endoscope, lung function test and cardiac ultrasound were prescribed according to symptoms, signs or a positive chest image.

*GI, gastrointestinal dysfunction, with symptoms or proved by endoscope; PF, pulmonary fibrosis, proved by image or lung function test (moderate to severe diffusion dysfunction); PAH, pulmonary artery hypertension, proved by cardiac ultrasound or chest image.

†Treatments before enrollment were listed according to a time sequence. HCQ, hydroxychloroquine; PA, penicillamine; MP, methylprednisolone; herbs, refer to Chinese traditional medications; Pred, prednisolone; LEF, leflunomide; TwHF, Tripterygium wilfordii Hook F, extract from a Chinese herb, proved to treat rheumatoid arthritis; Cyc, cyclophosphamide.

Table S2. Primers used in quantitative RT-PCR.

	forward	reverse
col1a1	TAAGGGTCCCCAATGGTGAGA	GGGTCCCTCGACTCCTACAT
col1a2	GTAACTTCGTGCCTAGCAACA	CCTTTGTCAGAATACTGAGCAGC
acta2	CCCAGACATCAGGGAGTAATGG	TCTATCGGATACTTCAGCGTCA
GAPDH	AGGTCGGTGTGAACGGATTTG	TGTAGACCATGTAGTTGAGGTCA

Table S3. Amplifying primers and mutation primers used in luciferase report assay.

	Forward	reverse
CK1α 3′ UTR	AAGGAAAAAAGCGGCCGCCATGAATTGTGGAAGAGAAGCAG	CCCAGATCGTATAGATGTTTACAGTCTCGAGCGG
CK1α 3′ UTR mutation	TACTAGATTCTCTTAGGACTCTATCGGCC	TTAATAGCAAGAACATTATAGAGGGCCGAT
SHIP1 3′ UTR	ATAAGAATGCGGCCGCGAAACGGCCTCACTTCTCTG	ACTGGGAAACACAGGGGATACCGCTCGAGCGG
SHIP1 3′ UTR mutation	TGGGTCCTGAGATGTTATTACATCCGACTA	TTAATAGCAAGAACATTATAGAGGGCCGAT
